# Does acute response to stellate ganglion block predict the success of cardiac sympathetic denervation? Preliminary evidence

**DOI:** 10.1016/j.hroo.2026.05.011

**Published:** 2026-05-22

**Authors:** Ioannis Doundoulakis, Luigi Pannone, Roberto Scacciavillani, Domenico Giovanni Della Rocca, Gezim Bala, Juan Sieira, Gian Battista Chierchia, Mark La Meir, Andrea Sarkozy, Carlo de Asmundis

**Affiliations:** 1 Heart Rhythm Management Centre, Postgraduate Program in Cardiac Electrophysiology and Pacing, Universitair Ziekenhuis Brussel – Vrije Universiteit Brussel, European Reference Networks Guard-Heart, Brussels, Belgium; 2 Cardiac Surgery Department, Universitair Ziekenhuis Brussel – Vrije Universiteit Brussel, Brussels, Belgium

**Keywords:** Stellate ganglion block, Cardiac sympathetic denervation, Sympathetic adrenergic activity, Ventricular tachycardia, Implantable cardioverter-defibrillator

**Figure:**
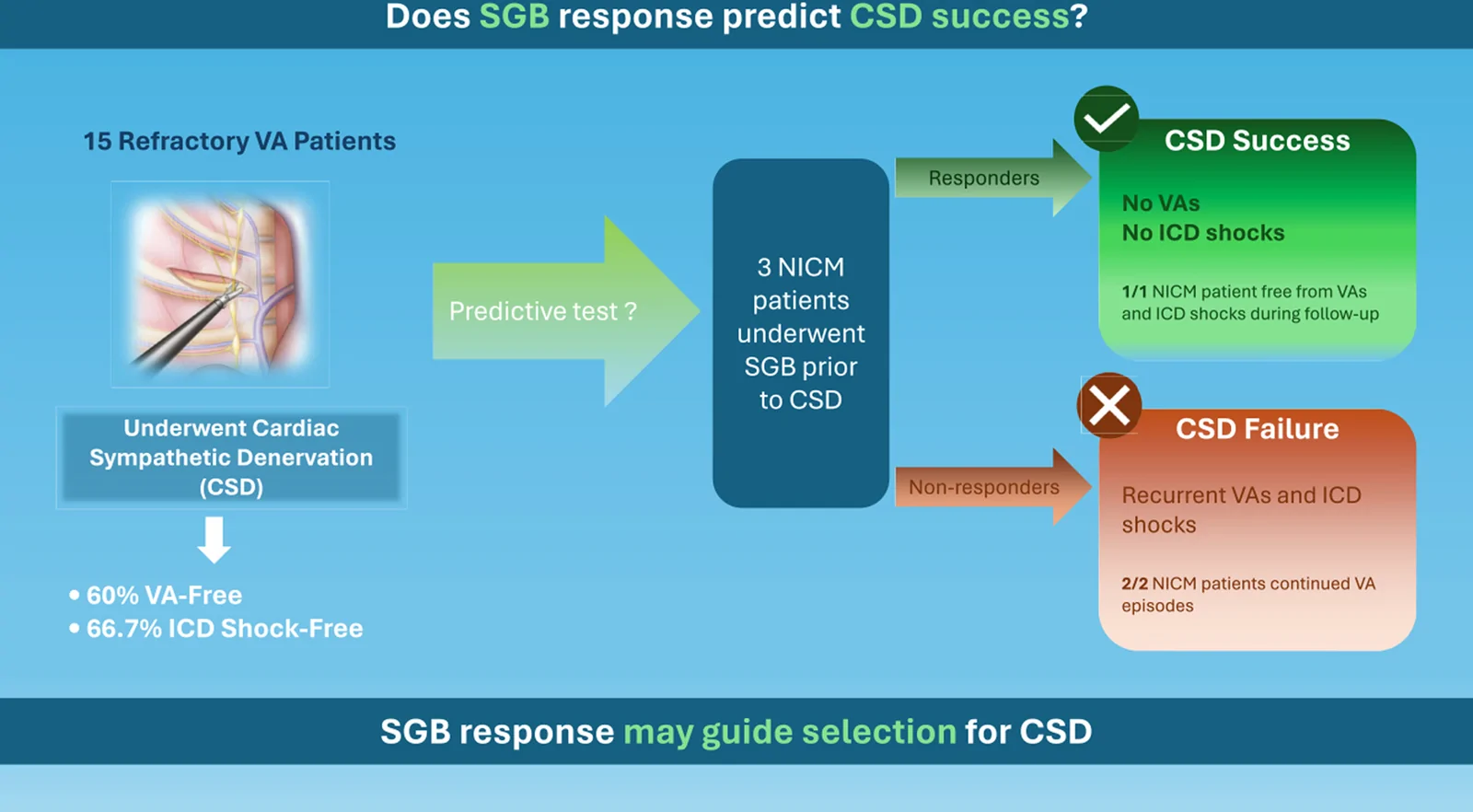

**Key Findings**

▪ This study shares the results of our experience with cardiac sympathetic denervation at a single high-volume European center. Freedom from ventricular arrhythmias was achieved in 60% of our sample.
▪ In patients with nonischemic dilated cardiomyopathy and refractory ventricular arrhythmias, a positive response to stellate ganglion block may predict sustained benefit from cardiac sympathetic denervation.
▪ This perfect concordance in our small nonischemic dilated cardiomyopathy subgroup supports the concept that stellate ganglion block can help identify cases in which sympathetic hyperactivity is a dominant driver amenable to permanent denervation.
▪ In contrast, nonresponders to stellate ganglion block may have alternative mechanisms less likely to benefit from cardiac sympathetic denervation, highlighting the potential value of stellate ganglion block as a minimally invasive selection tool.

Cardiac sympathetic denervation (CSD) has been shown to reduce sympathetic adrenergic activity and extend the duration of cardiac electrical action potentials.^1^ A recent combined analysis of studies showed that CSD resulted in a 60% rate of ventricular tachycardia not recurring over an average follow-up of approximately 15 months, alongside a notable decrease in the average number of implantable cardioverter-defibrillator (ICD) shocks per patient.^2^ Whether response to temporary stellate ganglion block (SGB) predicts long-term success of permanent CSD in patients with refractory ventricular arrhythmias (VAs) remains uncertain. Previous data suggest that SGB may serve as a diagnostic tool to identify sympathetic hyperactivity as a driver of VA, potentially guiding patient selection for CSD while avoiding unnecessary surgery in nonresponders. We hypothesized that acute VA suppression after SGB would correlate with freedom from VA and ICD shocks after surgical thoracoscopic CSD.^3^^,^^4^

We retrospectively analyzed 15 consecutive adult patients (mean age 41.8 ± 21.1 years, 33.3% female) who underwent CSD at our center between October 2015 and February 2020 for refractory VA despite optimized antiarrhythmic therapy and previous catheter ablation (mean 3.4 ± 1.8 procedures). All patients signed an informed consent that had been approved by our institutional review board. This study complied with the Declaration of Helsinki as revised in 2013 and was approved by the ethics committee. Underlying substrates included nonischemic dilated cardiomyopathy (NIDC) (n = 5), idiopathic ventricular fibrillation (n = 5), catecholaminergic polymorphic ventricular tachycardia (n = 2), long QT syndrome (n = 1), and Brugada syndrome overlap with long QT (n = 2). Left-sided CSD was performed in 2 patients and bilateral in 13, via video-assisted thoracoscopic surgery under general anesthesia. Follow-up (mean 80.3 ± 17.7 months) was conducted via periodic ICD interrogation. At long-term follow-up of 80.3 ± 17.7 months, 60% of patients remained free from any VA and 66.7% were free from ICD shocks (Table 1). No major CSD-related complications, Horner syndrome, or deaths occurred.

**Table 1:** Baseline characteristics and long-term outcomes after cardiac sympathetic denervation

Age	LVEF	Diagnosis	VA ablation	Stellate ganglion block	Type of CSD	Follow-up	
						ATP	Shock
57	50	IVF	5	-	Bilateral	-	-
75	25	NIDC	4	-	Bilateral	**+**	-
67	25	NIDC	6	-	Bilateral	**+**	**+**
63	35	NIDC	2	**+**	Bilateral	**+**	**+**
37	60	CPVT	5	-	Bilateral	-	-
19	65	IVF	1	-	Bilateral	**+**	**+**
20	65	IVF	2	-	Bilateral	-	-
14	65	CPVT	2	-	Left sided	-	**+**
67	35	NIDC	4	**+**	Bilateral	**+**	**+**
25	55	BRS + LQTS	2	-	Bilateral	-	-
30	55	LQTS	5	-	Left sided	-	-
57	50	IVF	5	-	Bilateral	-	-
16	60	BRS + LQTS	1	-	Bilateral	-	-
36	45	NIDC	4	**+**	Bilateral	-	-

Quantification of the ventricular arrhythmia burden (ATP and shocks) before SGB, in the interval between SGB and CSD, and after CSD for the 3 patients with NIDC who underwent SGB: patient 1, 63 years old (nonresponder) (5 tachytherapies/mo, 2 ATPs/mo, and 3 shocks/mo before SGB; 2 tachytherapies, 1 ATP, and 1 shock between SGB and CSD; and continued burden [2 tachytherapies/mo, 1 ATP/mo, and 1 shock/mo] after CSD); patient 2, 67 years old (nonresponder) (8 tachytherapies/mo, 3 ATPs/mo, and 5 shocks/mo before SGB; 6 tachytherapies, 3 ATPs, and 3 shocks between SGB and CSD; and persistent high burden [5 tachytherapies/mo, 2 ATPs/mo, and 3 shocks/mo] after CSD); and patient 3, 36 years old (responder) (6 tachytherapies/mo, 3 ATPs/mo, and 3 shocks/mo before SGB; 0 events between SGB and CSD; and 0 events after CSD).

ATP = antitachycardia pacing; BRS = Brugada syndrome; CPVT = catecholaminergic polymorphic ventricular tachycardia (n = 2); CSD = cardiac sympathetic denervation; IVF = idiopathic ventricular fibrillation; LQTS = long QT syndrome; LVEF = left ventricular ejection fraction; NIDC = nonischemic dilated cardiomyopathy; SGB = stellate ganglion block; VA = ventricular arrhythmia.

In a subgroup of 3 patients with NIDC who presented with electrical storm, ultrasound-guided left SGB was performed as acute therapy before CSD. SGB resulted in the immediate termination of the refractory electrical storm in 1 patient (with no complications), whereas it had no effect on the other 2. After CSD, the clinical outcomes mirrored the SGB response exactly: only the SGB responder remained free from recurrent VA and ICD shocks during long-term follow-up, whereas the 2 SGB nonresponders continued to experience VA episodes (detailed quantitative data on antitachycardia pacing and shocks before SGB, between SGB and CSD, and after CSD are presented in Table 1).

This study shares the results of our experience with CSD at a single high-volume European center. Freedom from VAs was achieved in 60% of our sample. Our research also demonstrates that, in patients with NIDC and refractory VA, a positive response to SGB (defined as acute suppression of electrical storm) may predict sustained benefit from CSD. This perfect concordance in our small NIDC subgroup supports the concept that SGB can help identify cases in which sympathetic hyperactivity is a dominant driver amenable to permanent denervation. In contrast, nonresponders to SGB may have alternative mechanisms less likely to benefit from CSD, highlighting the potential value of SGB as a minimally invasive selection tool.

Managing patients with VAs that do not respond to standard treatments and those experiencing frequent VA episodes is challenging. Increasing evidence suggests that CSD is an effective treatment option, especially for individuals with inherited channelopathies. Larger, prospective studies are needed to validate SGB as a predictor of CSD response across substrates and to refine patient selection for this adjunctive therapy in refractory VA.

## Disclosures

A.S. received research grants from Daiichi Sankyo and Bayer, has received speaker fees from Menarini and Bayer, and is a consultant for Biosense Webster, Medtronic, Biotronik, and MicroPort. G.B.C. received compensation for teaching purposes and proctoring from Medtronic, Abbott, Biotronik, Boston Scientific, and Acutus Medical. M.L.M. is a consultant for AtriCure. C.d.A. receives research grants on behalf of the center from Biotronik, Medtronic, Abbott, LivaNova, Boston Scientific, AtriCure, Philips, and Acutus. The other authors have no conflicts of interest to disclose.
